# Efficacy of a blended low-intensity internet-delivered psychological programme in patients with multimorbidity in primary care: study protocol for a randomized controlled trial

**DOI:** 10.1186/s12888-019-2037-3

**Published:** 2019-02-11

**Authors:** Alicia Monreal-Bartolomé, Alberto Barceló-Soler, Adoración Castro, Mª. Ángeles Pérez-Ara, Margalida Gili, Fermín Mayoral, Maria Magdalena Hurtado, Esperanza Varela Moreno, Cristina Botella, Azucena García-Palacios, Rosa M. Baños, Yolanda López-Del-Hoyo, Javier García-Campayo

**Affiliations:** 1 Primary Care Prevention and Health Promotion Research Network, RedIAPP, Carlos III Health Institute, Madrid, Spain; 2 Aragon Institute for Health Research, IIS Aragon, Zaragoza, Spain; 3 Primary Care Prevention and Health Promotion Research Network, RedIAPP, Madrid, Spain; 4 Institut Universitari d’Investigació en Ciències de la Salut, IUNICS-IDISPA, University of the Balearic Islands, Palma, Spain; 5 Mental Health Department, Institute of Biomedicine of Malaga, University Regional Hospital of Malaga, Malaga, Spain; 6 Jaume I University, Castellon de la Plana, Spain; 7 CIBER Physiopathology Obesity and Nutrition (CIBERobn) Carlos III Health Institute, Madrid, Spain; 8 Department of Clinical and Basic Psychology and Biopsychology, Faculty of Health Sciences, University Jaume I, Castellon, Spain; 9 Department of Psychological, Personality, Evaluation and Treatment, University of Valencia, Valencia, Spain; 10 Department of Psychology and Sociology, University of Zaragoza, Zaragoza, Spain; 11 Instituto de Investigación Sanitaria Aragón, Hospital Universitario Miguel Servet, Zaragoza, Spain

**Keywords:** Randomized controlled trial (RCT), Primary care, Multimorbidity, Depression, Information and communication technologies (ICTs)

## Abstract

**Background:**

The World Health Organization (WHO) has included comorbidity between depression and a chronic disease among the 10 leading global health priorities. Although there is a high prevalence of multimorbidity, health care systems are mainly designed for the management of individual diseases. Given the difficulty in delivering face-to-face psychological treatments, alternative models of treatment delivery have been proposed, emphasizing the role of technologies such as the Internet. The aim of this study is to assess the efficacy in Primary Care (PC) of a blended low-intensity psychological intervention applied using information and communication technologies (ICTs) for the treatment of multimorbidity in PC (depression and type 2 diabetes/low back pain) by means of a randomized controlled trial (RCT). Our main hypothesis is that improved usual care combined with psychological therapy applied using ICTs will be more efficacious for improvement in the symptomatology of multimorbidity, compared to a group with only improved treatment as usual six months after the end of treatment.

**Methods:**

A protocol has been designed combining a face-to-face intervention with a supporting online programme that will be tested by an RCT conducted in three different regions (Andalusia, Aragon and the Balearic Islands). The RCT will evaluate three hundred participants diagnosed with depression and type 2 diabetes/low back pain. Four highly experienced research groups specializing in clinical psychology are involved in this trial, and there will be ample possibilities for translation and transfer to usual clinical practice.

**Discussion:**

This clinical trial will lead to improvement in financial sustainability, maximizing the use of resources and responding to principles of efficiency and effectiveness. Furthermore, based on the evaluation of the feasibility of implementing this intervention in primary care facilities, we expect to be able to suggest the intervention for incorporation into public policy. In conclusion, positive results of this study could have a significant impact on one of the most important health-related problems, multimorbidity.

**Trial registration:**

ClinicalTrials.gov, NCT03426709. Registered retrospectively on 08 February 2018.

## Background

Multimorbidity, the presence of two or more medical conditions of long duration, is a prevalent finding, reaching levels of approximately 23% in European countries and increasing with age until it constitutes the norm in people aged 65 years and over [1]. However, the structure of health systems and those of medical research and education is designed for the prevention, treatment and management of isolated diseases and not multimorbidity.

The only meta-analysis to look at the efficacy of comorbidity treatment analysed 9 randomized controlled trials (RCTs), most of them in elderly patients [2]. The interventions consisted of a change in the provision of care (case management or interdisciplinary collaboration) or patient-oriented interventions. Information and communication technologies (ICTs) were not used in any of the cases. The results indicate that it is difficult to improve outcomes in this population, but those interventions that focus on specific risk factors or difficulties may be more effective. An important limitation in the literature is that no cost-effectiveness studies were included, nor was there any inclusion of qualitative studies attempting to understand the barriers in the interaction between health systems and patients [2]. In addition, changes in the provision of medical services are more complex and costly, making them less sustainable over time than personalized interventions. However, forms of personalized intervention based on ‘stepped care’ models and using ICTs have not been evaluated in multimorbidity.

Current guidelines for the treatment of comorbidity emphasize the need to personalize treatment, after an individualized evaluation of the disorders and their context, as well as to negotiate the therapeutic goals with the patients and re-evaluate them throughout the process, a process denominated the ‘Ariadne Principles’ [3]. All of this requires communication skills and time availability which not always possible in current health systems [4]. Therefore, complementary strategies are required to support general practitioners (GPs) in the provision of personalized and appropriate care to these patients [1].

The few studies published on multimorbidity in Spain, mainly in the elderly population, confirm that the negative impact on quality of life and disability is largely caused by mental disorders [5], more specifically depression [6]. This represents an important worldwide public health problem, as it is estimated that it will be the second leading cause of disability in the world by 2020 [7], with a very high economic cost [8]. Owing to its huge prevalence, 13.9–29% in primary care (PC) [9], international health authorities consider that available economic resources will be unable to meet the psychological treatment needs of this population [10]. For this reason, innovative and cost-effective alternatives that make use of ICTs for the treatment of depression and involve minimal face-to-face services are being proposed. The use of ICTs has proven effective both for depression and different mental health problems [11]. Therefore, prestigious organizations such as the National Institute of Clinical Excellence (NICE) in the UK have included in their guide [12] for online treatments such as Beating the Blues to treat depression.

Meta-analyses confirm that the two interventions of first choice for depression are pharmacotherapy and/or psychotherapy. The results are similar in the short term, but higher in the long term for psychological treatments, with lower dropout rates and fewer relapses [13, 14]. Different national and international clinical guidelines [12, 15] have proposed a stepped care model in PC, whereby a large proportion of patients are treated first with low-intensity interventions, with significant clinical benefits. In depression, low-intensity interventions are offered to patients with mild or moderate depressive symptoms.

The World Health Organization (WHO) has included comorbidity between depression and a chronic medical disease among the 10 leading global health priorities [5]. The comorbidity between depression and somatic illness means a significant increase in the disease burden since it implies a greater number of symptoms, higher morbidity, higher health care costs and a worse functioning and quality of life [16]. Current evidence supports a bidirectional relationship between depression and medical illness [17]. The suggested mechanisms to explain this complex relationship would include both biological aspects and behavioural variables. Depression is also associated with worse adherence to treatment in patients with comorbidity, for example, in antihypertensive or diabetes treatment [18, 19].

As regards medical conditions, although hypertension is highly prevalent (50–60%) [5], the most disabling conditions are osteoarticular diseases, diabetes and cerebral infarction [5, 6]. Therefore, the study will focus on two physical conditions comorbid to depression that involve the greatest disability, loss of quality of life and higher health costs: diabetes and chronic pain.

In 2013, the International Diabetes Federation (IDF) [20] reported that 382 million people in the world had diabetes, estimating that 592 million people will have diabetes by 2035, which would mean an increase of 55%. In terms of healthcare provision, it is estimated that in 2013, 548 billion dollars were spent worldwide on the care of diabetes and its complications, a rate that will increase over the years. The risk person with diabetes has of suffering from depression is approximately double that of a person without diabetes [21]. One in four patients with diabetes has depression [22], with higher mortality in these patients [23], and therefore depression and diabetes should be treated together and not as isolated diseases [23]. A review on the effectiveness of depression treatments in patients with diabetes [24] confirmed that it is effective, more specifically psychotherapy combined with self-care education for patients, with improvements shown in both psychological variables and glycaemic control.

Finally, the comorbidity of depression and chronic pain is extremely high in PC (56%) and reaches even higher rates in secondary care (50–69%) [25]. Given the high level of disability associated with the comorbidity of chronic pain and depression, and regardless of the direction of this association, national guidelines and consensus [15, 25] indicate the need to perform an objective assessment of pain in patients with depression by means of validated instruments. In addition, they establish that an integral therapeutic intervention should be considered, contemplating pharmacological and non-pharmacological treatments. The most common pain affects the lumbar skeletal muscle and is of mechanical origin, and almost the entire population suffers from it sometime in their life [26]. Together with depression, they lead the ranking of global disability caused by diseases [27]. Clinical guidelines recommend a treatment that includes education, physical exercise, physiotherapy and acupuncture [28]. However, there is not enough evidence on long-term results or on the superiority of any of these treatments over others [29–31]. A recent systematic review with meta-analysis has found long-term superiority of cognitive behavioural therapy compared to other therapies in relation to pain, disability and quality of life [32]. Despite the advantages that they could bring, there are no studies on the application of ICTs to either multimorbidity or any of the medical conditions included in this study [33].

The aim of this study is to evaluate the effectiveness in PC of a blended low-intensity psychological intervention applied through ICTs in the treatment of multimorbidity in PC (depression and type 2 diabetes / low back pain) in an RCT. Our main hypothesis is that the treatment-as-usual intervention, enhanced through the delivery of low-intensity psychological therapy by ICTs, will be more effective for the improvement of the symptomatology of multimorbidity in PC, in relation to a group with treatment-as-usual only, measured at 6 months after completion of the treatment.

## Methods

### Study design

This study is a two-armed and multicentre RCT, coordinated by the Aragon’s group. Participants will be randomly allocated to one of two conditions: a) low intensity Internet-delivered psychological intervention and b) improved treatment-as-usual (iTAU) groups in primary care.

### Setting and study sample

Participants will be recruited from PC health centres in the three Spanish regions participating in the study (Andalusia, Aragon and the Balearic Islands) from patients who meet the inclusion criteria and to whom the characteristics of the study will be explained. Patients will be recruited by GPs working in these PC centres until the required sample is completed, with a quota of 100 patients assigned to each centre. The inclusion and exclusion criteria are given in Table 1.

**Table 1:** Inclusion and exclusion criteria

Inclusion criteria	Exclusion criteria
Minimum age of 18 years	Any diagnosis of a disease that may affect the central nervous system (brain condition, traumatic brain injury, dementia, etc.)
DSM-5 diagnosis of Major Depression or persistent depressive disorder, mild or moderate depression expressed as a score lower than 14 in the Patient Health Questionnaire (PHQ-9)	Other psychiatric diagnoses or acute psychiatric illness (substance dependence or abuse, history of schizophrenia or other psychotic disorders, eating disorders, etc.), except for anxiety disorder or personality disorders
Ability to understand oral and written Spanish	Any medical, infectious or degenerative disease that may affect mood
Willingness to participate in the study and signing informed consent	Presence of delusional ideas or hallucinations whether consistent or not with mood
Duration of depressive symptoms 2 months or more	Suicide risk
Diagnosis of one of the following two conditions: - Type 2 Diabetes (Diagnosis according to criteria of the American Diabetes Association, ADA) - Lower back pain (Diagnosis of non-specific chronic low back pain according to the definition established by the CPG COST B-13 with a duration of at least 6 months)	
Possession of and ability to use a computer, an Internet connection and a mobile phone	

### Sample size

The sample size of this study was based on the possibility of detecting differences between the intervention programme versus treatment-as-usual, for a difference in the main variable of at least 0.5 standard deviations. This effect size was obtained from other studies, and it is considered a clinically relevant criterion [34, 35]. Assuming equal variance between the groups, a power of 80%, and a level of significance of 5%, 63 subjects were estimated for each group. To be able to compare the subjects treated with the specific intervention versus those receiving with treatment-as-usual only, as well as depending on the conglomerate to which they belong (each disease group), 4 groups were estimated with 63 subjects in each one, with a total of 252 subjects. An experimental mortality loss of 15% was estimated [36, 37], so the required size was finally 300 subjects (100 at each PC centre).

### Recruitment

Participants will be recruited in PC settings by participating GPs from patients fulfilling study criteria. When potential patients are identified by a GP, the characteristics of the study will be explained to them, and if the patients are interested in participating, they will be asked to sign an informed consent. The GPs will complete referral forms, indicating that the patients meet the criteria and will provide a brochure to present the study and an information sheet. The GPs will send the referrals and the patients’ signed consent forms by e-mail to the local researcher. The assessor researcher will contact the participants to agree on the established evaluation times. Some patients may prefer to postpone their decision to participate in the study, in which case they will be provided with information about the study by their GPs and how to contact the research team (by phone, email, or by leaving their information on the website).

The assessor researcher will clarify any doubts, ensure that the participants have read the information about the study, and make sure that they have understood the two experimental conditions. Then the assessor will determine their inclusion in the study from the psychological tests and biological variables related to the inclusion criteria before contacting an independent researcher to implement randomization. This researcher will be unaware of the characteristics of the study. Recruitment will be done consecutively until the final sample sizes are reached.

### Randomization, allocation and masking of study groups

At such a time, the assessor will collect the baseline data and contact a person independent of the research group to perform the individual randomization and inform the assessor of a code that corresponds to the type of treatment (unknown by the assessor) and send it to the researcher with the baseline data. Another independent person will do the data monitoring with a general practitioner. The randomization will be conducted by blocks of patients from the three PC centres, with 100 patients selected from each centre so that 150 patients are randomized into each of the two arms. Participants agree to their inclusion before finding out the treatment to which they will be allocated. All participants will be free to withdraw from the treatment at any time.

### Interventions

#### Internet-delivered psychological intervention

All interventions (except iTAU) comprise two face-to-face individual sessions and 6 online, individual and interactive therapeutic modules. Each face-to-face session will be 90 min in length. The online therapeutic modules are oriented to work on different psychological techniques, supported by multimedia material (videos, audio, etc.), as Internet support, and the duration of each module will be approximately 60 min.

The content of the modules is explained in depth later. The structure of the modules always follows the same scheme: each begins with the explanation of the contents of the module, different exercises are proposed, and then the self-test questions are presented to verify whether the explanation has been understood. If any user does not correctly answer any question, the computer program will immediately provide the correct feedback with a simple explanation. The module ends with the proposal of homework with the aim of practising what has been learned. In addition, before starting each module, a check is made to see whether the participants have carried out the proposed tasks and responds by congratulating them or encouraging them to do the task. It is very important to carry out these tasks in order to consolidate everything learned in the programme and so that the strategies offered by the programme are turned into skills. An important aspect is the possibility of reviewing the content of the different modules. The programme is designed to last 8–12 weeks. After some time without accessing the computer program (a time that can be programmed), the user will receive a reminder e-mail to continue completing the modules. The clinician can modify the period that should pass without entering to receive said reminder. The programme contents are the following:

- *Face-to-face session 1. Presentation of the programme:* Motivational techniques will be used to increase adherence of participants to face-to-face sessions and homework, and adherence to pharmacological treatment will be also promoted as one of the main focal points of the programme. The online computer program will be presented and patients will be trained in the procedure to log in and use it from home.
- *M1. Psychoeducation of depression:* The impact of depression on patients’ quality of life and functional ability, as well as on the prognosis of diabetes and other chronic diseases, will be described. The initial symptoms of depression and how to respond when they appear will be analysed. Self-control under stress will be emphasized, with patients taught to do relaxation exercises and to reward efforts and achievements made. In addition, specific techniques and very useful and practical tips to reduce stress in daily life will be explained.
- *M2. Healthy Life Style*: The need to carry out regular physical activity will be explained. The Mediterranean diet will be introduced, with education on the need to follow 6 healthy dietary commands. All of this will come with information and practical exercises on how to systematize food-related behaviours. Developing a social support network will be emphasized. People will be shown specific techniques on how to create and maintain an adequate social support network.
- *M3. Behavioural activation:* The importance of establishing and maintaining an adequate level of activity and involvement with life will be considered. Patients will be taught to schedule activities, and the need to monitor the performance of significant activities will be explained. The essential tool of this module will be the ‘Activity Diary’.
- *M4. Positive psychology:* This module will help patients to see the importance of positive emotions and learn procedures that generate positive experiences, encouraging involvement in pleasant and meaningful activities and contact with other people.
- *M5. Mindfulness and compassion*: This module will include components of Mindfulness-based Cognitive Therapy (MBCT). Core mindfulness practices will be carried out: the practice of breathing, the body scan and the 3-min exercise. The importance of establishing a regular mindfulness practice, as well as regular informal practice, will be considered. Some basic elements of compassion will be included, such as the self-compassionate pause.
- *Face-to-face session 2. Review of completed modules already and practice*. This is a structured session comprising: a) resolution of doubts, b) performing some of the most important practices (proposed to be done periodically at home), c) emphasizing the continuous putting into practice of the strategies learned and d) farewell and intervention closure.
- *M6. Relapse prevention and maintenance*: This module aims to strengthen the strategies learned during the programme. The possibility of continuing to practice the strategies learned once the treatment is completed will also be discussed, with emphasis on the fact that participants can continue to use the online computer program during the entire follow-up period (6 months) for this purpose. It will also teach participants how to identify and cope with future high-risk situations. Finally, instructions will be given to conduct follow-up evaluations.

This group of patients will also receive iTAU from their GPs, as described in the next paragraph.

#### iTAU at primary care level

All the patients included in the study (whether they receive the Internet-delivered psychological intervention or not) will be also treated by their GPs. In practice, treatment-as-usual in PC is any kind of treatment administered by the GP to the patient with depression. However, this treatment in primary care will be improved because the participating GPs will receive a training programme on the widely used Spanish Guide for the Treatment of Depression in Primary Care, which is based on the NICE guidelines on subject [15]. In case of suicide risk, severe social dysfunction or worsening of symptoms, it is recommended that patients are referred to mental health facilities [38]. For both groups, the use of health and social services – including consultations with professionals, pharmaceutical use and other resources, will be registered using the Client Service Receipt Inventory (CSRI) [39].

### Instruments

The assessor will be blind to the type of treatment that will be administered to patients. In addition, this assessor will be different from the person who collects the measurements of the study results. As far as possible, GPs will also be blind to the intervention arm to which each patient is allocated, since their intervention should be based only on usual practice, based on the criteria set out in the Guide for the Treatment of Depression in Primary Care.

**Fig. 1:**
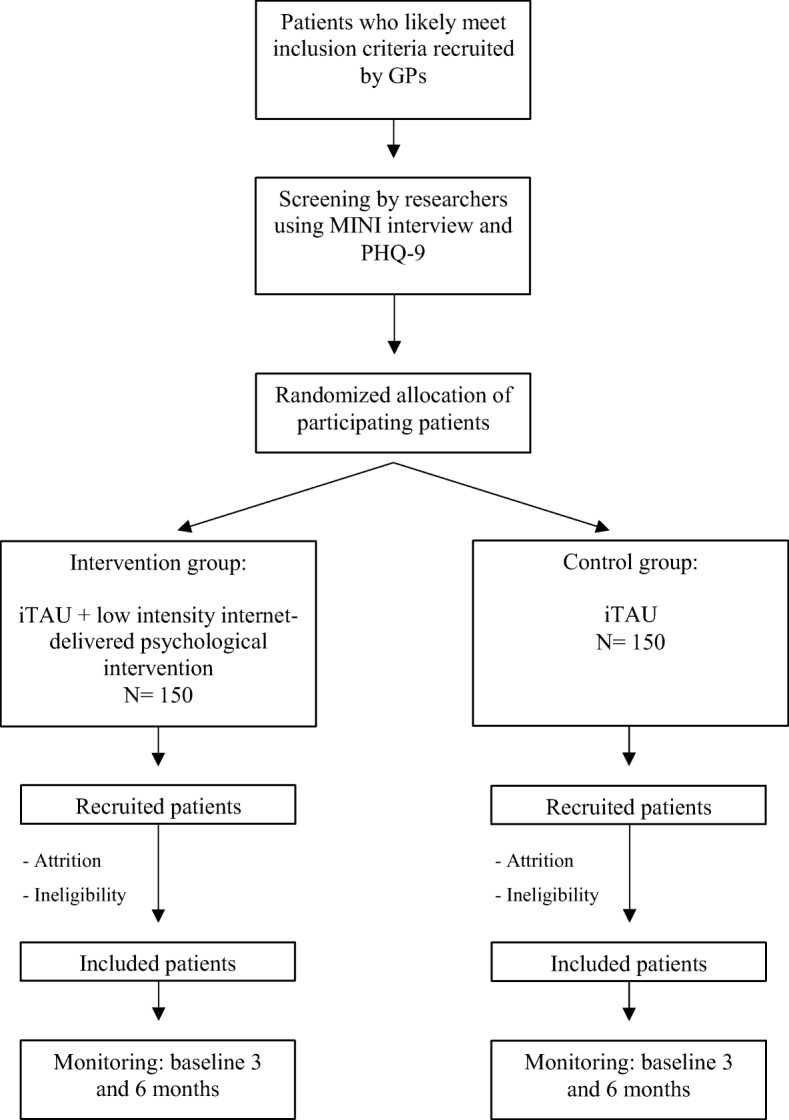
Flowchart of the study: Randomization, sampling and monitoring of patients

Patients will be assessed at baseline (post-randomization), after the treatment, and at 3-month and 6-month follow-ups, in order to test whether the improvements achieved during the therapy are maintained in the long term. The study variables assessed are summarized in Table 2. The study flowchart is shown in Fig. 1.

**Table 2:** Study variables

Instrument	Assessment area	Time to assessment	Applied by
PHQ-9 (40, 41)	Severity of depression	Baseline and follow-up sessions^a^	Researcher A phone Online (follow-up sessions^a^)
Glycosylated haemoglobin	Diabetes control	Baseline and follow-up sessions^a^	Online
FPS-R [42]	Pain intensity	Baseline and follow-up sessions^a^	Online
Roland-Morris Scale [43–46]	Physical limitation	Baseline and follow-up sessions^a^	Online
MINI [47]	Psychiatric diagnosis	Baseline	Researcher A phone
Sociodemographic data	Gender, age, marital status, education, occupation, economical level	Baseline	Researcher A phone
SF-12 Health Survey [48, 49]	Health-related quality of life	Baseline and follow-up sessions^a^	Online
CRSI [39, 50]	Health and social services use	Baseline and 6 months post-treatment	Researcher A phone (Baseline) Researcher B phone (6 months post-treatment)
EQ-5D-3 L [51, 52]	Quality of life related to health	Baseline and follow-up sessions^a^	Online
OFS [53]		Baseline and follow-up sessions^a^	Online

*PHQ-9* Patient Health Questionnaire, *FPS-R* Faces Pain Scale –Revised, *MINI* Mini-International Neuropsychiatric Interview, *CSRI* Client Service Receipt Inventory, *EQ-5D-3 L* EuroQol-5D-3 L, *OFS* Openness to the Future Scale

^a^Follow-up sessions: post-treatment, 3 and 6 post-treatment months

#### Main outcome

According to the objectives of the study, the primary outcome will be a composite that considers the following variables:

Depressive symptom severity will be assessed via the Patient Health Questionnaire (PHQ-9) [40] using the Spanish validated version [41]: Percentage of patients with reduction ≥2 points. The PHQ-9 [40], validated Spanish version [41] is one of the questionnaires that assess the intensity of depression most widely used in pharmacological and psychological studies. It is a brief and self-applied scale whose objective is to help diagnose depression (DSM-IV criteria) and determine its severity. Similarly, it is also useful to monitor changes experienced by patients over time. It will be taken four times: at baseline, post-treatment, and at 3-month and 6-month follow-ups.

Control of diabetes will be measured by VR d = glycosylated haemoglobin (% ≤ 7 or % with a decrease of 0.50 baseline), HbA1c, fasting capillary blood glucose, weight (kg), height (cm), waist circumference (cm), and abdominal diameter (cm).

Pain intensity and physical limitation will be measured. Pain intensity will be assessed via the Faces Pain Scale – Revised (FPS-R) [42], a self-report measure in which the patient scores the chosen face with 0, 2, 4, 6, 8, or 10, counting from left to right, where 0 = no pain, and 10 = very much pain. Disability will be assessed via Roland-Morris Scale [43]: This scale is designed to reliably determine the level of physical disability resulting from non-specific low back pain. In this regard, ‘physical disability’ is defined as the limitation in the performance of daily activities between 0 (absence of disability for back pain) and 24 (maximum possible disability). A change in score only has clinical relevance if it is of 2 or more points, although the optimal threshold is between 3 and 4 [44–46]. VR d = % with decrease ≥2 points.

The composite would be weighted to give 1/3 or 1/2 to each of the main measures of depression, diabetes and pain, obtaining an aggregate score. The calculation of the weighting of the composite variables would be performed by means of multiple regression.

#### Secondary outcomes

##### Sociodemographic variables

The following sociodemographic data will be collected: gender, age, marital status (single, married/long-term relationship, separated/divorced, widowed), education (years of education), occupation and economical level.

##### Mini-International Neuropsychiatric interview (MINI)

This is a short structured diagnostic psychiatric interview that yields key DSM-IV and ICD-10 diagnoses [47]. The MINI can be administered in a short period of time and clinical interviewers only need brief training. The MINI has been translated into Spanish and the Spanish version validated [47]. This psychiatric interview will allow the diagnosis of depression to be made at baseline in the study.

##### SF-12 Health Survey

This is an instrument that measures the state of perceived health. It consists of 12 items that measure 8 dimensions. The calculation of the scores is based on reference standards, on a scale of 0 (worst possible health status) to 100 (best possible health status). The SF-12 has good psychometric qualities [48] and is validated in Spanish [49].

##### CSRI Spanish version [39, 50]

Questionnaire for collecting information about the use of health and social care services and other economic impacts (such as time off work due to illness). The variant used in this study was designed to collect retrospective data on service utilization during the 12 previous months.

##### EuroQol-5D-3 L (EQ-5D-3 L) [51]

The utility scores were obtained from the EuroQol-5D (EQ-5D) classification system, which measures health-related quality of life on a scale of 0 (as bad as death) to 1 (perfect health). However, this two-part index may also provide negative values that correspond to health states perceived as worse than death. Part 1 records self-reported problems in each of five domains: mobility, self-care, usual activities, pain/discomfort and anxiety/depression. Part 2 records the subject’s self-assessed health on a visual analogue scale (VAS), a 10-cm vertical line along which the best and worst imaginable health states are scored between 100 and 0, respectively. The utility scores for these health states were assigned using the Spanish population rates [52].

##### Openness to the Future Scale (OFS) [53]

This 10-item instrument measures positive affectivity towards the future that can be a prospective protective factor for mental health and an indicator of psychological adjustment. The scale presents good psychometric properties in a Spanish sample.

### Ethical aspects

Informed consent will be obtained from the participants before they are aware of the group to which they are to allocated. Before they give their consent, patients will be provided with a general overview of the aims and characteristics of the study and the psychological and pharmacological intervention. Patients in the iTAU arm will be allowed to take the psychological intervention programme at the end of the study for ethical reasons. The study has been developed according to national and international standards (Helsinki and Tokyo Convention). Given that the interventions involve the use of the Internet, an important ethical issue is data protection. *Advanced Encryption Standard* (AES) strategies regarding data encryption and use of personal passwords will be implemented in order to guarantee the protection of personal information. The data will be treated anonymously and will only be used for the purposes of the study. The confidentiality of the participants included in the study will be guaranteed. Finally, the study protocol was approved by the Research Ethics Committee of each autonomous community: (CEICA in Aragon, CEIC in the Balearic Islands and the Regional Ethics and Research Committee of the province of Malaga, Andalusia).

### Analysis strategy

#### Analysis of clinical efficacy

Intention-to-treat and per-protocol analysis will be used. Analysis will include the description and ‘head-to head’ comparison between the two groups. Descriptive statistics of the included variables (mean and 95% confidence interval for normally distributed quantitative variables; and median and interquartile range for abnormally distributed quantitative variables) will be performed. To confirm the main hypothesis, all variables will be compared (t0-tk) using an analysis of variance (ANOVA) and post-hoc tests or Kruskal-Wallis non-parametric tests. Finally, more sophisticated multivariate analysis, including multilevel regression, will be used. The effect size of improvement and the number needed to treat (NNT) in each arm will be estimated.

#### Descriptions of costing procedure

Data collection on the use of health and social services will be collected through the CSRI-Spanish version [39, 50]. Costs of the study will be estimated from the healthcare and societal perspectives during the previous six months (before baseline), and during the six months of follow-up. Direct health care costs will be calculated by adding the costs derived from pharmacological consumption, use of health services and clinical tests, and costs incurred by the staff running the intervention.

The cost of medication will be calculated by determining the price per milligram during the study, according to the databases of the Spanish Ministry of Health and Consumer Affairs, including value-added tax. The total cost of medication will be calculated by multiplying the price per milligram by the daily dose in milligrams and the number of days of treatment. The main source of the unit cost data for medical tests and health services is the OBLIKUE database of health care costs [54]. The OBLIKUE database contains information about Spanish health service costs and is derived by systematic reviews of the literature. Indirect costs (lost productivity) will be calculated considering sick leave days and multiplying them by the minimum daily wage in Spain for 2018. Finally, total costs will be calculated by adding direct and indirect costs. The unit costs will be expressed in euros (€), based on 2018 prices.

The two interventions in this study will be conducted by government agencies and financed by the Carlos III Health Institute; their implementation will not incur additional costs for the Spanish National Health Service. Therefore, costs associated with the interventions in the study will not be included.

The economic evaluation of this study will follow the general guidelines for conducting pharmacoeconomic analyses in Spain. It will also follow the Guidelines of the International Society for Pharmacoeconomics and Outcomes Research (ISPOR) [55] and the Consolidated Health Economic Evaluation Reporting Standards (CHEERS) [56].

#### Utility scores

Utility scores will be obtained from two health-related quality of life questionnaires EuroQol 5D and SF-12. QALYs will be calculated based on these scores using the Spanish EQ-5D tariffs [57]. Along with EQ-5D utility scores, scores recorded on the EQ VAS will also be used as an outcome for the analysis.

The perspective used in our analysis will be twofold: the social perspective, to include all costs and quality of life, and the perspective of the Spanish health system.

The time horizon of the study is six months, so it will not be necessary to apply a discount factor to the costs.

#### Cost-utility analysis

Cost-effectiveness will be explored through the calculation of incremental cost-effectiveness ratios (ICERs) for the intervention group using the iTAU group as the control. To estimate the ICER (ratio between incremental costs and incremental effectiveness) the following equation will be used:

$$ ICER=\frac{Cost\kern0.17em active\kern0.17em intervention\ group\hbox{-} Cost\ control\ intervention\ group}{Effectiveness\ or\ Utility\ active\ intervention\ group\hbox{-} Effectiveness\ or\ Utility\ control\ intervention\ group\ } $$

Cost-utility will be explored through the calculation of incremental cost-utility ratios (ICURs), which are defined as the ratio between incremental costs and incremental effects measured on QALYs [58]. QALYs will be approximated by using the area-under-the-curve technique.

Data collection will be performed using Excel software and statistical analysis will use SPSS (SPSS Inc., Chicago, IL, USA) software version 20, licensed by the University of Malaga.

Frequency and proportions will be used for descriptive statistics of categorical or qualitative variables. For quantitative variables, the mean and standard deviation will be obtained.

Inferential statistical analysis will use the Chi-square test for qualitative variables, one-way ANOVA test for qualitative and quantitative variables and Student’s t-test for quantitative variables. In all cases, statistical significance will correspond to a value of *p* < 0.05.

### Forecast execution dates

Initial recruitment of patients: September 2018.

Finalization of patient recruitment: April 2019.

Finalization of patient monitoring period: April 2020.

Publication of results: October 2020.

## Discussion

The presence of multimorbidity makes it difficult for patients to seek help, receive a conclusive diagnosis, receive quality care and adherence to treatment. The present study proposes the evaluation by means of a leading and novel intervention in the field of ICTs of disorders of high prevalence and producing great disability, such as the multimorbidity between depression and type 2 diabetes/chronic low back pain. In addition, in a PC setting, where these patients with a great need for healthcare provision are increasingly attended. The aim is to evaluate the effectiveness and cost-effectiveness of this treatment in the management of depression with medical comorbidity, as well as to achieve maximum impact for the Spanish National Health System, generating knowledge that can be included in the clinical guidelines for multimorbidity treatment in PC and throughout the health system.

It should be noted that this is the first study of its kind to use ICTs in the treatment of multimorbidity. It is also the first treatment for multimorbidity in Spain. This study has several strengths: it follows the guidelines of comorbidity treatment (Ariadne’s Principles); it will be conducted on a wide age range – most similar studies have been carried out on a geriatric population, but non-geriatric patients will also be included, facilitating preventive and health promotion aspects. In order to overcome one of the limitations described in meta-analyses preformed on previously published works, it includes a cost-effectiveness study; finally, the results obtained from this study can be easily transferred to the health system through the modification of the current management given to this group of conditions.

No particular difficulties are expected as regards the recruitment of patients with depression or their participation in the study. The negative attitudes of some GPs may hinder their recommending this treatment, therefore a training session on the study will be held at the participating PC health centres for professionals interested in participating. The main limitation of the study may be a significantly greater rate of dropouts in the treatment groups (40% in previous studies). Efforts will be made to maintain dropout rates in the range of 30% in order to overcome this limitation. In any case, an associated qualitative study will be included with this study to analyse the barriers and limitations of this therapy on Spain’s public health system. On the other hand, in addition to the intention-to-treat analysis, an analysis per protocol will be performed to determine the efficacy of the intervention in those patients who do not abandon the treatment.

The treatment programmes used in this study includes therapeutic strategies (mindfulness, healthy lifestyle habits, positive psychology and behavioural activation) that have proven their efficacy for depression, type 2 diabetes and chronic low back pain treatment [13, 14, 23, 24, 28]. Furthermore, this intervention will lead to improved financial sustainability, maximizing the use of resources and responding to principles of efficiency and effectiveness. In conclusion, the positive results of this study could have a significant impact on one of the most important problems in the health context: multimorbidity.

## Abbreviations

ADA: diagnosis according to criteria of the American Diabetes Association
AES: Advanced Encryption Standard
CHEERS: Consolidated Health Economic Evaluation Reporting Standards
CSRI: Client Service Receipt Inventory
EQ-5D-3 L: EuroQol-5D-3 L
GPs: general practitioners
ICERs: incremental cost-effectiveness ratios
ICTs: information and communication technologies
ICURs: incremental cost-utility ratios
IDF: International Diabetes Federation
ISPOR: International Society for Pharmacoeconomics and Outcomes Research
iTAU: improved treatment-as-usual
MBCT: Mindfulness-Based Cognitive Therapy
MINI: Mini-International Neuropsychiatric Interview
NICE: National Institute of Clinical Excellence (UK)
NNT: number needed to treat
PC: primary care
RCT: randomized controlled trial
WHO: The World Health Organization
